# Sticky bacteria: Combined effect of galactose and high ferric iron concentration on extracellular polymeric substances production and the attachment of *Acidithiobacillus ferrooxidans* on a polymetallic sulfide ore surface

**DOI:** 10.3389/fmicb.2022.951402

**Published:** 2022-09-12

**Authors:** Eduardo A. Moncayo, Alexis Debut, Karla Vizuete, Diana Jumbo-Flores, Paulina Aguirre

**Affiliations:** ^1^Maestría en Química Aplicada, Facultad de Ciencias Exactas y Naturales, Universidad Técnica Particular de Loja, Loja, Ecuador; ^2^Grupo de Investigación y Desarrollo de la Biotecnología BioSin-Biociencias, Quito, Ecuador; ^3^Centro de Nanociencia y Nanotecnología, Universidad de las Fuerzas Armadas ESPE, Sangolquí, Ecuador; ^4^Grupo de Investigación en Materiales y Ambiente, Departamento de Química, Universidad Técnica Particular de Loja (UTPL), Loja, Ecuador

**Keywords:** attachment, *Acidithiobacillus ferrooxidans*, inducers, ferric iron, galactose

## Abstract

Adaptation and microbial attachment mechanisms for the degradation of sulfide ores are mediated by the production of extracellular polymeric substances (EPS) and their role in biofilm formation. EPS production responds to induction mechanisms associated with environmental conditions. In this study, the double induction of EPS with galactose and high ferric iron concentrations in planktonic cells of *Acidithiobacillus ferrooxidans*, and their attachment on the surface of a polymetallic sulfide ore from Bella Rica-Azuay in Ecuador were evaluated. *A. ferrooxidans* cells were previously adapted to different concentrations of galactose [0, 0.15, and 0.25% (w/v)], using two ferrous iron concentrations as an energy source (9 and 18 g L^–1^) in a 9K culture medium. EPS production and its effect on mineral attachment were determined at the time point of maximal growth. The results obtained show a maximum cell attachment of 94.1% within 2 h at 0.15% of galactose and 18 g^⋅^L^–1^ of ferric iron concentration, compared to 71.4% without galactose and 9 g^⋅^L^–1^ of ferric iron. The maximum concentration of EPS was obtained with a 0.25% galactose concentration; however, it did not result in greater attachment compared to 0.15% galactose concentration. Through the combined induction of low galactose concentration and high ferric iron concentration, the percentage of bacterial attachment can be increased and, therefore, a possible increase in the rate of biooxidation and bioleaching could be obtained.

## Introduction

Biooxidation and bioleaching processes allow to obtain the metals from low-grade sulfide ores (Srichandan et al., 2019) and have been widely accepted in the mining industry when conventional treatments are not economically feasible (Kaksonen et al., 2020). Biooxidation and bioleaching are carried out by acidophilic chemolithoautotrophic microorganisms capable of oxidizing reduced sulfur and/or iron compounds to obtain energy (Sajjad et al., 2019). *Acidithiobacillus ferrooxidans (A. ferrooxidans)* is the most characterized acidophilic bacterium due to its potential to oxidize iron and sulfur, with studies focused on specific genomic and metabolic features (Hödar et al., 2012; Campodonico et al., 2016; Latorre et al., 2016).

During biooxidation and bioleaching, microorganisms oxidize Fe^2+^ to Fe^3+^. The last one acts as an oxidizing agent, chemically oxidizing metal sulfides, reducing them to Fe^2+^ during mineral dissolution (Vera et al., 2013). The role of microorganisms is to regenerate the oxidizing agent Fe^3+^ (Rawlings and Johnson, 2007). This mechanism occurs at the interface between the microorganism and the mineral; in this process, the presence of extracellular polymeric substances (EPS) is required, which allows bacterial attachment to produce a biological attack on the mineral surface, forming a biofilm (Li et al., 2016).

In *A. ferrooxidans*, initial adhesion is mediated by EPS and type IV pili (Sand and Gehrke, 2006; Li et al., 2010). Type IV pili and EPS contain adhesins and outer membrane proteins that allow them to colonize the mineral surface (Yu et al., 2011; Tu et al., 2013). The functional groups of the EPS generate hydrophobic forces and electrostatic interactions that hold cells together with the mineral, contributing to the global stability of biofilm matrices (Li et al., 2016). The composition and quantity of EPS differ according to the type of substrate and energy source, in which the cells are cultured; thus, the attachment mechanisms also differ depending on the mineral surfaces (Li et al., 2016). In addition, it has been observed that the EPS composition of cells cultured on minerals, such as pyrite, is very similar to the EPS composition of cells cultured in the planktonic phase in a ferrous sulfate-containing medium, but with a lower amount of EPS (Gehrke et al., 1998; Rohwerder and Sand, 2007; Vu et al., 2009).

With the discovery of an alternative metabolic pathway that allows *A. ferrooxidans* to use galactose as a carbon source for EPS production (Barreto et al., 2005), strategies for inducing EPS in the presence of this sugar have been carried out, obtaining positive results (Bellenberg et al., 2012; Pavez et al., 2013; Saavedra et al., 2013b,^2020^). Furthermore, it has been observed that adapting *A. ferrooxidans* to high ferric ion concentrations produces greater amounts of EPS (Saavedra et al., 2013a), and, in the same way, the EPS produced by induction with galactose also allows *A. ferrooxidans to* tolerate high ferric ion concentrations (Saavedra et al., 2020). However, when biooxidizing bacteria, an excess of EPS has not always resulted in a greater attachment (Aguirre et al., 2017, 2018). With this background, being *A. ferrooxidans* the predominant microorganism in the microbial consortia of biooxidation cells, this study aimed to increase the bacteria attachment capacity to a refractory gold-bearing polymetallic sulfide ore by regulating the EPS production with both high-iron concentrations and galactose to increase its biooxidation potential.

## Materials and methods

### Polymetallic sulfide ore

The polymetallic sulfide ore used in the attachment assays was from Bella Rica, Azuay-Ecuador. X-ray diffraction analysis (Profile and structure analysis performed with TOPAS software of the equipment Bruker D8-Advance) shows the following minerals in percentages (%): 30.24 quartz, 34.26 pyrite, 0.41 chalcopyrite, 0.95 galena, 3.41 sphalerite, 15.19 orthoclase, 8.24 gypsum,0.89 apatite-(Sr-OH), 2.41 pyrrhotite, 2.61 arsenopyrite,0.92 cobalt pentlandite, and 0.46 magnetite. The ore was ground to a particle size of 75 μm, verified by the D80 of the ASTM#200 sieve.

### Culture, adaptation, and growth kinetics of *Acidithiobacillus ferrooxidans*

The *A. ferrooxidans* strain used in this study was obtained from the biomining and bioprocess laboratory of Universidad Técnica Particular de Loja. Bacterial adaptation was carried out through successive cultures for 2 months in six treatments corresponding to the planned experiments (9 g^⋅^L^–1^ of Fe^2+^ supplemented with 0, 0.15, and 0.25% galactose, and 18 g^⋅^L^–1^ of Fe^2+^ with 0, 0.15, and 0.25% galactose). *A. ferrooxidans* was grown at 30°C and 180 rpm in 250 ml flasks with 50 ml of modified 9K culture medium (composition in g^⋅^L^–1^: KCl 0.004; MgSO_4_ 7H_2_O 0.005; (NH_4_) H_2_PO_4_ 0.15; CaCl_2_ 0.0012) supplemented with FeSO_4_^⋅^7H_2_O for the treatments with 9 g^⋅^and 18 g^⋅^L^–1^ of ferrous iron, respectively. The pH was adjusted to 1.8 with H_2_SO_4_. The microorganisms were considered adapted when they achieved a cell number equivalent to the non-galactose treatments, and they completed the Fe^2+^ oxidation time that remained similar and constant in each iron concentration treatment. After adaptation, growth kinetics were determined for each condition from an initial inoculum of 1^⋅^10^7^ cells^⋅^ml^–1^ in 9K medium at pH 1.8 by cell count and Fe^2+^ consumption. Each culture was followed until the Fe^2+^ concentration was close to zero, corresponding to iron oxidation greater than 90%. Cell numbers were determined with a Neubauer chamber of 0.02 μm of depth every 3 h. Fe^2+^ consumption, Fe^3+^ production, and total iron amount were determined at each point using the ferrozine method (Lovley and Phillips, 1987). The maximum specific growth rate (μ_max_), the yield of biomass (Y_X/S_), and the volumetric productivity (P_v_) were calculated with the Equations (1)–(3), respectively, in the exponential growth phase.


(1) $$\mu_{m\,a\,x}=\frac{L\,n\,X-L\,n\,X_{0}}{t}$$

(2) $$Y_{x/s}=\frac{\Delta \,X_{m}}{-\Delta \,S}$$

(3) $$P_{v}=\frac{\Delta \,\left[F\,e^{3+}\right]}{\Delta \,t}$$

Where X is the cell concentration achieved at the end of the exponential growth phase (cell^⋅^ml^–1^); *X*_*0*_ is the initial cell concentration at the beginning of the exponential growth phase (cell^⋅^ml^–1^); X_m_ is the cell mass concentration (g^⋅^L^–1^); [Fe^3+^] is the ferric iron concentration (g^⋅^L^–1^); S is the Fe^2+^ concentration (g^⋅^L^–1^); t is the time (h); Δ represents the increment of the variable from the initial stage to the final stage, and Ln represents the natural logarithm. The Fe^3+^ generation rate (σ_P_) was calculated as the slope of the Fe^3+^ concentration over time plot in the exponential growth phase.

### Production and extraction of extracellular polymeric substances

Adapted microorganisms were cultured with 9 and 18 g^⋅^L^–1^ of Fe^2+^, supplemented with 0, 0.15, and 0.25% of galactose. The EPS extraction was performed at the time point of maximal growth when the Fe^2+^ concentration reached zero. EPS was extracted using DOWEX cation exchange resin (DOWEX™ Marathon C) as it is a technique that does not cause cell damage to the cultures, avoiding breaking microorganism membranes (Tapia et al., 2013). A proportion of 70 g of resin per gram of cell dry weight was used, taking as reference the 1.2^⋅^10^–12^ g/bacteria and the microbial count. The resin was previously hydrated with 20 ml of PBS buffer at pH 7 under stirring for 1 h. Approximately 40 ml of culture were centrifuged at 5,800 *g* for 10 min discarding the supernatant. The cells were resuspended on the hydrating DOWEX resin. Cell suspension was shaken at 100 rpm and 4°C for 4 h. Subsequently, the extract was centrifuged at 4,500 g for 5 min and the supernatant was filtered through a 0.2-μm membrane. The filtered extracts were dialyzed in 3.4 kDa membrane tubes for 24 h in ddH_2_O water. Protein and carbohydrate contents were quantified. The cell suspension precipitate was cultured in a 9K medium with 9 g^⋅^L^–1^ of Fe^2+^ and, in addition, a sample of microbial cultures was analyzed for the absorption spectrum carried out over the whole wavelength spectrum from 325 to 825 nm, before and after EPS extraction to verify that there was no cell lysis during extraction (Tapia et al., 2013).

### Determination of carbohydrates and proteins in extracellular polymeric substances

Carbohydrates were determined by the sulfuric acid phenol method (Dubois et al., 1956), using glucose as the standard. Approximately 0.5 ml of 5% phenol was added to 1 ml of EPS extract, followed by 2.5 ml of concentrated sulfuric acid, and then mixed rapidly. The solution was cooled at room temperature for 10 min avoiding direct light exposure and then incubated at 30°C in a water bath for 30 min. The absorbance was read at 492 nm. The protein content in the EPS extract was determined by the bicinchoninic acid (BCA) method (Walker, 2009), using the Thermo Scientific™ Pierce™ BCA Protein Determination Kit.

### Extracellular polymeric substances observation by transmission electron microscopy

The presence/absence of EPS was evaluated using a Transmission Electron Microscope (TEM, FEI, TECNAI, and G2 Spirit Twin). A drop (5 μl) of bacterial suspension obtained at the time point of maximal growth of each assay was deposited on a formvar/carbon-coated TEM-grid (300-mesh). Then, samples were negatively stained using phosphotungstic acid (PTA) 0.5% for 1 s. The TEM micrographs were obtained at 80 kV by using a TEM equipped with an Eagle 4k HR camera.

### Microbial attachment assays

The bacterial attachment was determined by cell counting in the liquid phase and contrasted by qPCR using DNA extracted from the ore sample. Bacterial attachment assays were performed in 250 ml Erlenmeyer flasks with 100 ml of 9K culture medium, supplemented with 2% w/v of ore previously acidified to pH 1.8, with sulfuric acid under a nitrogen atmosphere to prevent chemical oxidation, and sterilized at 105°C for 24 h in dry heat. The mineral medium was inoculated with cells at peak growth time at concentrations of 1^⋅^10^8^ cells^⋅^ml^–1^ and incubated for 7 h at 30°C and 180 rpm. Samples of 5 ml were taken at time intervals of 1, 10, 30, 60, 120, 180, 240, and 420 min. The samples were centrifuged at 6 *g* for 3 min and the number of bacteria in the liquid phase was determined by counting in a Neubauer chamber under a phase contrast microscope. The attachment percentage was determined by the difference between the initial number of inoculated cells and the number of cells remaining in the liquid phase. Attachment kinetics was contrasted by real-time PCR (qPCR) from the DNA extracted from the mineral phase of the samples at 1, 30, 60, and 180 min; each assay was performed in triplicate.

### DNA extraction and qPCR

Attachment kinetics by qPCR was performed by quantifying the number of copies of DNA extracted from 0.1 g of ore, which is equivalent to 5 ml of mineral in suspension at 2% w/v. About 5 ml of ore suspension were taken at times of 1, 30, 60, and 180 min from the attachment assays and were centrifuged at 6 g for 3 min. The supernatant was immediately discarded. DNA of the cells attached to the precipitated ore was extracted using the FastDNA™ Spin Kit for Soil-MP Biomedicals, eluting in 50 μl of nuclease-free water. The qPCRs were performed with the primers designed by Aguirre et al. (2021): 5′-TCTTCGGATGCTACAG-3′ and 5′-CGSGTTACBTACACACT-3′, which amplify a fragment of 785 base pairs corresponding to the region 674-1450 of the 16S ribosomal RNA gene of *A. ferrooxidans*, using the EvaGreen qPCR Mastermix-ABM. The DNA copy number standards were obtained by PCR from DNA extracted from pure cultures of *A. ferrooxidans*, according to the specifications of the Wizard^®^ Genomic DNA Purification Kit—Promega using the GoTaq^®^ Green Master Mix—Promega, and subsequent PCR product purification using the Wizard^®^ SV Gel and PCR Clean-Up System—Promega kit. The attachment percentage was determined by the difference between the initial cell number inoculated per gram of ore and the number of attached cells per gram of ore (number of DNA copies per gram of ore).

### Statistical analysis

Differences between EPS production and attachment percentages were evaluated by ANOVA and subsequent Bonferroni’s *post hoc* test using the statistical software R version 4.1.1 (Supplementary material). Each experiment was performed in triplicate by analytical duplicate (*n* = 6).

## Results

### Microbial adaptation and growth parameters

As galactose is not used for the growth of *A. ferrooxidans* since it causes inhibition (Saavedra et al., 2020), a laboratory adaptation process was carried out through successive cultures for concentrations of 9 and 18 g^⋅^L^–1^ of iron in the presence of 0.15 and 0.25% of galactose. After 2 months of adaptation, the growth kinetics of *A. ferrooxidans* was evaluated at different concentrations of iron and galactose. Table 1 summarizes the kinetic parameters obtained.

**TABLE 1:** Kinetic parameters of *A. ferrooxidans* cultured at different concentrations of iron and galactose.

Treatment	Fe^2+^ (g^⋅^L^–1^)	Galactose (% w/v)	Max cell count (cell^⋅^ml^–1^)	μ *_*max*_* (h^–1^)	σ_p_ (h^–1^)	Y_X/S_ (g_cell_/g_Fe_^2+^)	P_v_ (g^⋅^L^–1⋅^h^–1^)
Af9	9	0	1.48^⋅^10^8^ ± 5.46^⋅^10^6^	0.14 ± 0.004	0.68 ± 0.03	0.019 ± 0.001	0.62 ± 0.01
Af9015	9	0.15	1.63^⋅^10^8^ ± 7.24^⋅^10^6^	0.13 ± 0.01	0.77 ± 0.01	0.021 ± 0.003	0.65 ± 0.01
Af9025	9	0.25	1.57^⋅^10^8^ ± 1.06^⋅^10^7^	0.14 ± 0.01	0.68 ± 0.01	0.020 ± 0.001	0.66 ± 0.02
Af18	18	0	2.85^⋅^10^8^ ± 1.23^⋅^10^7^	0.09 ± 0.001	0.64 ± 0.01	0.019 ± 0.011	0.66 ± 0.01
Af18015	18	0.15	2. 93^⋅^10^8^ ± 7.81^⋅^10^6^	0.08 ± 0.002	0.70 ± 0.01	0.019 ± 0.011	0.66 ± 0.01
Af18025	18	0.25	2.77^⋅^10^8^ ± 1.16^⋅^10^7^	0.09 ± 0.002	0.72 ± 0.01	0.019 ± 0.010	0.67 ± 0.003

Af9, 9 g^⋅^L^–1^ of iron and 0% of galactose; Af9015, 9 g^⋅^L^–1^ of iron and 0.15% of galactose; Af9025, 9 g^⋅^L^–1^ of iron and 0.25% galactose; Af18, 18 g^⋅^L^–1^ of iron and 0% of galactose; Af18015, 18 g^⋅^L^–1^ of iron and 0.15% of galactose; Af18025, 18 g^⋅^L^–1^ of iron and 0.25% galactose; μ_max_, maximum specific growth rate; σ_P_, product (Fe^3+^) generation rate; Y_X/S_, yield of biomass; P_v_, Volumetric productivity. The data correspond to the mean values of triplicates ± SD (n = 6).

*A. ferrooxidans* cultured with galactose exhibited a similar cell count compared with the treatments cultured without galactose for each iron concentration at the same period. Table 1 shows similar growth parameters for the different galactose concentration treatments denoting a complete adaptation. The volumetric productivity for Fe^+3^ remained relatively constant comparing 9 and 18 g^⋅^L^–1^ iron treatments.

### Effect of galactose and ferric iron on the production of extracellular polymeric substances

**FIGURE 1:**
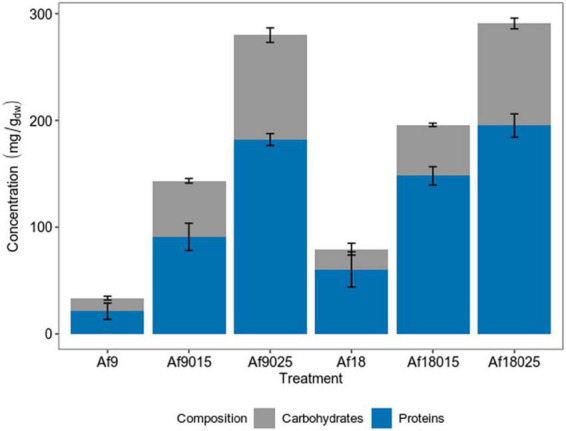
Carbohydrates and protein compositions of the EPS of *A. ferrooxidans* cultured at different concentrations of iron and galactose. Blue color: protein; Gray color: Carbohydrates. The data correspond to the mean values of triplicates ± SD (*n* = 6).

EPS extractions were performed at peak growth time for each treatment, corresponding to 24 h for 9 g^⋅^L^–1^ iron culture, and 36 h for 18 g^⋅^L^–1^ iron culture. Total EPS production was quantified considering the amount of protein and carbohydrates (Figure 1).

Figure 1 shows that EPS production increased, along with galactose concentration, for both 9 and 18 g^⋅^L^–1^ ferric iron treatments, reaching a higher concentration in cells cultured with 0.25% of galactose.

The media of EPS concentrations were 33.39, 143.61, and 280 mg/g_dw_ for the induction with 9 g^⋅^L^–1^ of iron and 0, 0.15%, and 0.25% of galactose, respectively, and 83.81, 195.82, and 290.90 mg/g_dw_ for the induction with 18 g^⋅^L^–1^ of iron and 0, 0.15%, and 0.25% of galactose, respectively. For 9 g^⋅^L^–1^ iron treatment, the EPS production increased 4.3 and 8.3 times in cells cultured with 0.15 and 0.25% of galactose; whereas, for 18 g^⋅^L^–1^ iron treatment, the EPS production increased 2.5, 5.8, and 8.7 times for cells cultured with 0,0.15, and 0.25% of galactose compared with the assays with 9g^⋅^L^–1^ without galactose.

Table 2 shows the composition of the EPS in terms of the carbohydrate and protein content for each induction treatment. Data shows a higher concentration of carbohydrates and proteins in the inductions with 0.25% of galactose, followed by the 0.15% treatments. The ratio of proteins per amount of carbohydrates in EPS remained relatively constant in the treatments with 9 g^⋅^L^–1^ of iron with increasing concentration of galactose, being 1.74 for the treatment without galactose, 1.72 and 1.86 for the induction with 0.15 and 0.25% of galactose. In 18 g^⋅^L^–1^ iron treatment, the protein/carbohydrate ratio was 3.1 and 3.2 for the induction with 0 and 0.15% of galactose, decreasing to 2.04 in the induction with 0.25% of galactose.

**TABLE 2:** Carbohydrates and proteins composition of the extracellular polymeric substances.

Treatment	Extraction time (h)	(mg/g_dw_)			
		
		Carbohydrates	Proteins	Total EPS	Prot/carb
*Af9*	24	12.19 ± 1.80	21.19 ± 7.54	33.38 ± 5.95	1.74
*Af9015*	24	52.65 ± 2.17	90.95 ± 12.78	143.61 ± 11.84	1.72
*Af9025*	24	97.83 ± 6.77	182.16 ± 5.56	280.0 ± 12.31	1.86
*Af18*	36	18.91 ± 3.38	60.55 ± 16.39	79.46 ± 19.46	3.20
*Af18015*	36	47.57 ± 1.59	148.24 ± 8.53	195.81 ± 6.94	3.12
*Af18025*	36	95.66 ± 5.00	191.25 ± 10.91	290.90 ± 13.25	2.04

The data correspond to the mean values of triplicates ± SD (n = 6).

Comparing the EPS protein and carbohydrate content in the 18 g^⋅^L^–1^ iron treatment with the 9 g^⋅^L^–1^ iron treatment, it was observed that, for non-galactose assays, carbohydrates did not change significantly (*p* > 0.05), while protein content increased by 2.2 times. For 0.15 and 0.25% galactose assays, the carbohydrate content remained relatively constant, while the protein content, for the induction with 0.15% galactose, increased by 1.6 times and remained constant in the induction with 0.25% galactose. There was also no significant difference (*p* > 0.05) between the protein content comparing the treatments with 9 g^⋅^L^–1^ Fe and 0.15% galactose vs. the treatment with 18 g^⋅^L^–1^ iron and 0% galactose.

#### Extracellular polymeric substances visualization

**FIGURE 2:**
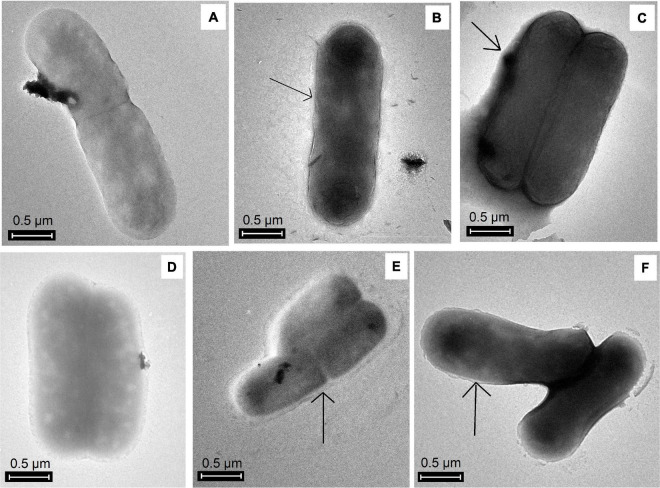
TEM images of EPS production by *A. ferrooxidans* in planktonic phase induced with different concentrations of iron and galactose. **(A)** Af9, **(B)** Af9015, **(C)** Af9025, **(D)** Af18, **(E)** Af18015, **(F)** Af18025. Black arrows show the presence of EPS.

EPS production in the planktonic phase was evidenced by TEM. Figure 2 shows that galactose-induced cells were surrounded by EPS (black arrows), whereas, in non-galactose treatments, there was no notable EPS visualization.

### Effect of extracellular polymeric substances induced with galactose and Fe^3+^ on the attachment of *Acidithiobacillus ferrooxidans* to the polymetallic sulfide ore

**FIGURE 3:**
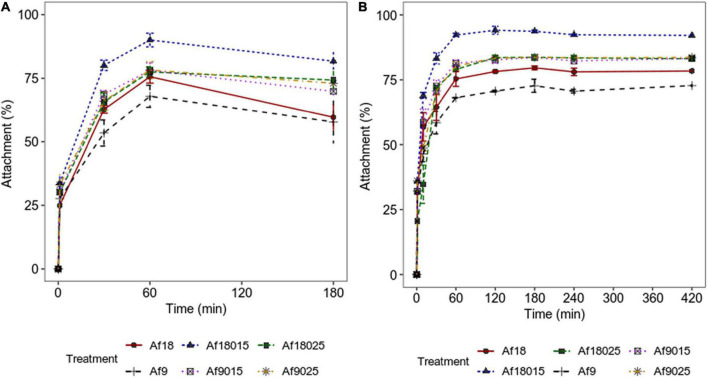
Attachment kinetics of *A. ferrooxidans* to a polymetallic sulfide ore. **(A)** Data obtained by qPCR. **(B)** Data obtained by counting cells in the planktonic phase using a Neubauer chamber (0.02 mm). Culture modified 9K medium supplemented with 2% polymetallic sulfide ore. The data correspond to the mean values of triplicates ± SD (*n* = 6).

The attachment of *A. ferrooxidans* is more significant in the first hours of biooxidation (Rodríguez et al., 2003); thus, the assays were performed during the first 7 h. The results of bacterial attachment during the first 3 h (qPCR) and 7 h (cell count) of biooxidation are shown in Figure 3.

**FIGURE 4:**
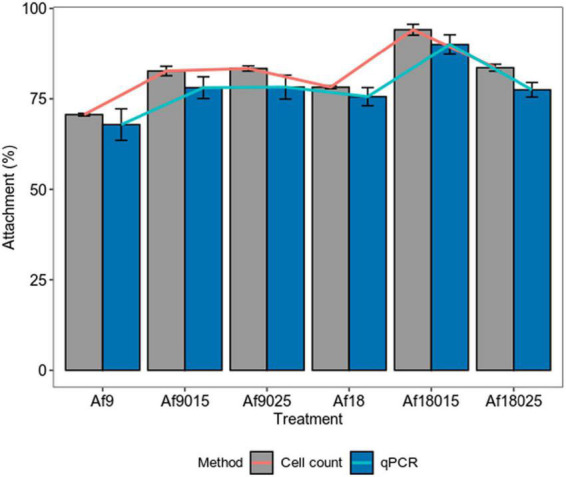
Maximum attachment percentage of *A. ferrooxidans* induced with different concentrations of iron and galactose to a polymetallic sulfide ore. Bars represent the maximum attachment percentage and lines represent the increase in attachment compared to the control assay (Af9). The data correspond to the mean values of triplicates ± SD (*n* = 6).

Figure 3 shows how galactose and high-iron concentrations increase the attachment of *A. ferrooxidans* to the ore in the first hours of the process. The maximum attachment percentages achieved are shown in Figure 4.

Figure 4 shows that for 9 g^⋅^L^–1^ iron treatment, the attachment percent increased by 12.3 and 12.9% measured by cell count, 10.3 and 10.4% as determined by qPCR for the induction, with 0.15 and 0.25% of galactose, respectively, compared to the control assays. On the other hand, in the induction with 18 g^⋅^L^–1^ of iron, the attachment percentage increased by 7.81, 23.7, and 13.1% measured by cell count, and 7.7, 22, and 9.6% determined by qPCR for the induction with 0, 0.15, and 0.25% of galactose, respectively. The maximum attachment percentage values reached were 70.4, 82.7, 83.3, 78.21, 94.1, and 83.5% measured by cell count, and 67.9, 78.2, 78.3,75.6, 89.9, and 77.5% determined by qPCR for the treatments Af9, Af9015, Af9025, Af18, Af18015, and Af18025, respectively, which suggest a higher attachment to the ore in case of a double induction in the production of EPS, with 0.15% galactose and 18 g^⋅^L^–1^ of iron.

## Discussion

It has been reported that chemolithoautotrophic microorganisms, including *A. ferrooxidans*, show considerable sensitivity to organic compounds, such as galactose, which may result in a growth inhibitory effect (Shafia and Wilkinson, 1969; Tuovinen and Kelly, 1973; Saavedra et al., 2013b,^2020^; Aguirre et al., 2018). Glucose is not necessarily inhibitory, but it may allow for the growth of *A. ferrooxidans* in the absence of its inorganic energy source (Tabita and Lundgren, 1971). However, through successive cultures, a strain of *A. ferrooxidans* adapted to 0.15 and 0.25% galactose concentrations were obtained without observing a reduction of its maximum specific growth rate (μ_max_), with values within the ranges reported for kinetics of *A. ferrooxidans* in similar defined culture media, compared with the one used in this study that was between 0.07 and 0.14 h^–1^ (Braddock et al., 1984; Boon et al., 1999; Bryan et al., 2012; Saavedra et al., 2020); this implies a complete adaptation to galactose. Despite the increase in the energy source, the volumetric productivity, with respect to biomass and Fe^3+^ generation, remained relatively constant in treatments cultured with 9 and 18 g^⋅^L^–1^ of Fe^2+^, along with the rate of substrate consumption; thus, increasing only the total Fe^2+^ oxidation time, which results in doubling the microbial count in 18 g^⋅^L^–1^ of Fe^2+^ treatments compared to 9 g^⋅^L^–1^ of Fe^2+^ treatments.

Barreto et al. (2005) identified genes related to galactose metabolism (Leloir pathway) directed to the EPS production; EPS are part of the biofilm. A rapid biofilm formation on pyrite has been reported when *A. ferrooxidans* were exposed to D-galactose and glucose (Bellenberg et al., 2012), favoring the biooxidation of pyrite (Pavez et al., 2013). In addition, it has been observed that the cultivation of *A. ferrooxidans* at low concentrations of galactose increases the production of EPS, and it allows the bacteria to tolerate higher concentrations of Fe^3+^ (Saavedra et al., 2020). In the present study, a gradual increase in both carbohydrate and protein EPS contents was observed as galactose concentration increased. EPS presence visualized by TEM was only remarkable in galactose-induced cells. The maximum EPS yield was achieved in the inductions with 0.25% galactose with 9 and 18 g^⋅^L^–1^ of iron, without observing a significant difference (*p* > 0.05) between these two treatments. Saavedra et al. (2020) observed a higher production of EPS when *A. ferrooxidans* was grown with 0.35% galactose, but with a reduction in the cell density due to its sensitivity to carbohydrates; thus, it is counterproductive to cultivate *A. ferrooxidans* at concentrations higher than 0.25% galactose.

The double induction with iron and galactose in the EPS production was evidenced in cells cultured with 0.15% galactose mainly in the protein content, in which, protein composition increased by 1.6 times in 18 g^⋅^L^–1^ iron treatments compared with 9 g^⋅^L^–1^ iron cultured cells, with no significant increase in the carbohydrate content. While, in non-galactose treatments, the single effect of Fe^3+^ could be evidenced, where the protein content increased by 2.8 times, whereas the carbohydrate content remained relatively constant (*p* > 0.05). This variation in the EPS content suggests that the composition of EPS depends on the stress mechanisms applied to *A. ferrooxidans* when adapting to the environment. Culture conditions directly influence the EPS content of biooxidizing bacteria, and it has been observed that when cells are grown on soluble substrates, they produce fewer amounts of EPS compared to solid substrates (Gehrke et al., 1998; Harneit et al., 2006; Rohwerder and Sand, 2007; Vu et al., 2009). However, the EPS content in the planktonic phase can be increased in biooxidizing bacteria using soluble substances, such as galactose and ferric ion (Aguirre et al., 2018, 2021).

The attachment and biofilm formation are mediated by EPS and required during the ore biooxidation process (Li et al., 2016). EPS functional groups allow bacteria to interact with minerals through electrostatic interactions and hydrophobic forces, holding cells together and contributing to biofilm stability (Li et al., 2016). Attachment in biooxidizing bacteria occurs during the first hours of contact with the mineral (Rodríguez et al., 2003; Vardanyan et al., 2013), observing in such studies a maximum attachment percentage in the first 2 h of mineral contact in all treatments. However, the percentage of attachment to the mineral varied depending on the induction treatment in the planktonic phase. For the 9 g^⋅^L^–1^ iron without galactose treatment, a maximum attachment of 70.4 ± 0.3% was achieved with an EPS content of 27.44 ± 5.95 mg/g_dw_, while in induction with 0.15 and 0.25% galactose, the attachment percentage increased to 82.7 ± 3 and 83.3 ± 3.3% for EPS concentrations of 90.95 ± 12.78 and 182.16 ± 5.56 mg/g_dw_, respectively. Curiously, in 18-g^⋅^L^–1^ iron induction, the percentage of attachment was 78.2 ± 0.4% without galactose, while in induction with 0.15% galactose, it increased to a maximum of 94.1 ± 2.7 and only 83.5 ± 0.9% in the induction with 0.25%, for EPS contents of 60.55 ± 16.39, 148.24 ± 8.53, and 191.23 ± 10.25 mg/g_dw_, respectively. Although the amount of carbohydrates and lipids maintain biofilm stability, proteins play a fundamental role in the attachment (Tu et al., 2013). During cell attachment, outer membrane proteins from EPS and adhesins (Pili-associated proteins) molecularly interact irreversibly with the functional groups of the mineral, keeping cells attached (Li et al., 2010; Chandraprabha and Natarajan, 2013; Alfaro-Saldaña et al., 2019). When comparing the composition of the EPS in 0.15% galactose-induced treatments, it is observed that the proteins increased by 60% for the induction with 18 g^⋅^L^–1^ of iron compared to the induction with 9 g^⋅^L^–1^ (Table 2), suggesting a positive effect on attachment with protein increase. Such percentage of attachment is not verified in 0.25% galactose treatments, where the composition of proteins and carbohydrates do not vary significantly. On the other hand, when comparing the 0.15% galactose and 18 g^⋅^L^–1^ iron treatments, with 0.25% galactose, 9 and 18 g^⋅^L^–1^ iron treatments, the amount of carbohydrates was doubled for the inductions, with 0.25% galactose, and the proteins remain just a little above in the 0.25% galactose treatments. However, this high amount of carbohydrate results in less attachment compared to 0.15% galactose and 18 g^⋅^L^–1^ iron induction. Similar results were reported in previous work for *A. thiooxidans*, which reached a higher attachment with 0.15% galactose, compared with 0.25 and 0.35% galactose induction, with equally increasing concentrations of EPS according to galactose concentration (Aguirre et al., 2017). Those results indicate that excess EPS results are not always in higher attachment and that the combined induction of galactose and iron could modulate the EPS production to maximize the attachment.

## Conclusion

Through the combined induction of galactose and ferric iron, the production of EPS in *A. ferrooxidans* was improved. The effect of the Fe^3+^ ion was mainly reflected in the protein content of EPS, while galactose increased the content of carbohydrates and proteins, suggesting that the biochemical composition and the number of EPS produced in *A. ferrooxidans* vary depending on the applied stress conditions. Despite an excess of EPS production, it did not maximize cell attachment. In this study, a higher attachment percentage (94%) was obtained for double induction with 18 g^⋅^L^–1^ of Fe^3+^ and 0.15% galactose, compared to treatments induced with 0.25% galactose (83.2% for 9 g^⋅^L^–1^ of Fe^3+^ and 83.5% for 18 g^⋅^L^–1^ of Fe^3+^) whose EPS carbohydrate content was almost double.

## Data availability statement

The original contributions presented in this study are included in the article/Supplementary material, further inquiries can be directed to the corresponding author/s.

## Author contributions

EM and PA: conceptualization and formal analysis. EM, DJ-F, AD, and KV: methodology. PA and DJ-F: resources and funding acquisition, writing, and reviewing. EM: writing the original draft preparation. All authors have read and agreed to the published version of the manuscript.
