# Supplementary material for: Sticky bacteria: Combined effect of galactose and high ferric iron concentration on extracellular polymeric substances production and the attachment of Acidithiobacillus ferrooxidans on a polymetallic sulfide ore surface
Source: Front Microbiol. 2022 Sep 12;13:951402. doi: 10.3389/fmicb.2022.951402 (PMC9512070; doi:10.3389/fmicb.2022.951402)
Supplement: Supplementary file 1 [file Data_Sheet_1.docx]

Supplementary Material

# Statistical analysis for EPS composition and Attachment percent of *A. ferrooxidans* induced with different concentration of iron and galactose

Differences between EPS production (Table 2) and maximum attachment percentages (Table S1) of *A. ferrooxidans* to a polymetallic mineral were evaluated by ANOVA and subsequent Bonferroni's post-hoc test using the statistical software R version 4.1.1. Each experiment was performed by triplicate with analytical duplicate. Table S2 shows the p-values obtained by ANOVA and Table S3 the p-values obtained by compare between each treatment by Bonferroni's post-hoc test.

**Table S1.** Maximum attachment percentages of *A. ferrooxidans*

| **Treatment** | **Culture conditions** | **Attachment (%)** | | **Increase compared to the control group (%)** | |
| --- | --- | --- | --- | --- | --- |
|  |  | **Cell count** | **qPCR** | **Cell count** | **qPCR** |
| *Af9** | 9 gL^-1^ Fe^2+^, 0%gal | 70,4 ± 0.3 | 67.9 ± 4.4 | - | - |
| *Af9015* | 9 gL^-1^ Fe^2+^, 0.15%gal | 82.7 ± 1.3 | 78.2 ± 3.0 | 12.3 | 10.3 |
| *Af9025* | 9 gL^-1^ Fe^2+^, 0.25%gal | 83.3 ± 0.7 | 78.3 ± 3.3 | 12.9 | 10.4 |
| *Af18* | 18 gL^-1^ Fe^2+^, 0%gal | 78.2 ± 0.4 | 75.6 ± 2.5 | 7.81 | 7.7 |
| *Af18015* | 18 gL^-1^ Fe^2+^, 0.15%gal | 94.1 ± 1.5 | 89.9 ± 2.7 | 23.7 | 22 |
| *Af18025* | 18 gL^-1^ Fe^2+^, 0.15%gal | 83.5 ± 0.9 | 77.5 ± 2.0 | 13.1 | 9.6 |

Af9* = Control group

**Table S2.** Mean comparison by ANOVA of EPS content and attachment percent of *A. ferrooxidans* induced with different concentration of iron and galactose

| **Total EPS content** | | | | | | | | | | |
| --- | --- | --- | --- | --- | --- | --- | --- | --- | --- | --- |
| **Factor** | **Df** | | **SS** | | **MS** | | **F** | | **p-value** | |
| Total EPS | | 5 | | 164814 | | 32963 | | 212.7 | | <0.0001 |
| Residuals | | 12 | | 1860 | | 155 | |  | |  |
| **Carbohydrates content in EPS** | | | | | | | | | | |
| **Factor** | **Df** | | **SS** | | **MS** | | **F** | | **p-value** | |
| Carbohydrates | 5 | | 20036 | | 4007 | | 258.9 | | <0.0001 | |
| Residuals | 12 | | 186 | | 15 | |  | |  | |
| **Protein content in EPS** | | | | | | | | | | |
| **Factor** | **Df** | | **SS** | | **MS** | | **F** | | **p-value** | |
| Proteins | 5 | | 73158 | | 14632 | | 123.3 | | <0.0001 | |
| Residuals | 12 | | 1423 | | 119 | |  | |  | |
| **Attachment percent by cell count** | | | | | | | | | | |
| **Factor** | **Df** | | **SS** | | **MS** | | **F** | | **p-value** | |
| Cell Count | 5 | | 883.0 | | 170.60 | | 186.2 | | <0.0001 | |
| Residuals | 12 | | 11.4 | | 0.95 | |  | |  | |
| **Attachment percent by cell qPCR** | | | | | | | | | | |
| **Factor** | **Df** | | **SS** | | **MS** | | **F** | | **p-value** | |
| qPCR | 5 | | 1515.4 | | 303.09 | | 26.97 | | <0.0001 | |
| Residuals | 30 | | 337.1 | | 11.24 | |  | |  | |

**Table S3.** p-values of mean comparison between treatments by Bonferroni's post-hoc test of the EPS content and attachment percent of *A. ferrooxidans* induced with different concentrations of iron and galactose

| **Total EPS content** | | | | | | | | |
| --- | --- | --- | --- | --- | --- | --- | --- | --- |
|  | **Af18** | **Af18015** | | **Af18025** | | | **Af9** | **Af9015** |
| **Af18015** | <0.0001 | - | | - | | | - | - |
| **Af18025** | <0.0001 | <0.0001 | | - | | | - | - |
| **Af9** | 0.01028 | <0.0001 | | <0.0001 | | | - | - |
| **Af9015** | 0.00058 | 0.00370 | | <0.0001 | | | <0.0001 | - |
| **Af9025** | <0.0001 | <0.0001 | | 1.00000 | | | <0.0001 | <0.0001 |
| **Carbohydrates content in EPS** | | | | | | | | |
|  | **Af18** | **Af18015** | | **Af18025** | | | **Af9** | **Af9015** |
| **Af18015** | <0.0001 | - | | - | | | - | - |
| **Af18025** | <0.0001 | <0.0001 | | - | | | - | - |
| **Af9** | 0.87 | <0.0001 | | <0.0001 | | | - | - |
| **Af9015** | <0.0001 | 1.00000 | | <0.0001 | | | <0.0001 | - |
| **Af9025** | <0.0001 | <0.0001 | | 1.00000 | | | <0.0001 | <0.0001 |
| **Protein content in EPS** | | | | | | | | |
|  | **Af18** | **Af18015** | | **Af18025** | | | **Af9** | **Af9015** |
| **Af18015** | <0.0001 | - | | - | | | - | - |
| **Af18025** | <0.0001 | 0.00289 | | - | | | - | - |
| **Af9** | 0.01241 | <0.0001 | | <0.0001 | | | - | - |
| **Af9015** | 0.07625 | 0.00048 | | <0.0001 | | | <0.0001 | - |
| **Af9025** | <0.0001 | 0.03697 | | 1.00000 | | | <0.0001 | <0.0001 |
| **Attachment percent by cell count** | | | | | | | | |
|  | **Af18** | | **Af18015** | | **Af18025** | **Af9** | | **Af9015** |
| **Af18015** | <0.0001 | | - | | - | - | | - |
| **Af18025** | 0.00031 | | <0.0001 | | - | - | | - |
| **Af9** | <0.0001 | | <0.0001 | | <0.0001 | - | | - |
| **Af9015** | 0.00172 | | <0.0001 | | 1.00000 | <0.0001 | | - |
| **Af9025** | 0.00045 | | <0.0001 | | 1.00000 | <0.0001 | | 1.00000 |
| **Attachment percent by qPCR** | | | | | | | | |
|  | **Af18** | | **Af18015** | | **Af18025** | **Af9** | | **Af9015** |
| **Af18015** | <0.0001 | | - | | - | - | | - |
| **Af18025** | 1.00000 | | <0.0001 | | - | - | | - |
| **Af9** | 0.00612 | | <0.0001 | | 0.00040 | - | | - |
| **Af9015** | 1.00000 | | <0.0001 | | 1.00000 | 0.00017 | | - |
| **Af9025** | 1.00000 | | <0.0001 | | 1.00000 | 0.00014 | | 1.00000 |
